# The patient experience of relapsed refractory multiple myeloma and perspectives on emerging therapies

**DOI:** 10.1002/cnr2.1603

**Published:** 2022-02-15

**Authors:** Rebecca Crawford, Katharine S. Gries, Satish Valluri, John Fastenau, Ross Morrison, Tzu‐Min Yeh, Yunsi Olyslager, Jenna D. Goldberg, Jordan M. Schecter, Carolyn C. Jackson, William Deraedt, Lynda Doward

**Affiliations:** ^1^ RTI Health Solutions Manchester UK; ^2^ Janssen Global Services, LLC Raritan NJ USA

**Keywords:** immunotherapy, multiple myeloma, qualitative research, treatment choices

## Abstract

**Background:**

Relapsed refractory multiple myeloma (RRMM) is a disease that is nonresponsive or progressive on therapy, and although patients can achieve remission, relapse is common. As more treatment options become available for multiple myeloma (MM), it is important to understand patients' experiences of current and emerging therapies.

**Aims:**

This study aimed to better understand patient experiences with treatment and therapies for MM using qualitative interviews and patient‐reported information (PRI) shared on social media.

**Methods:**

Semistructured qualitative interviews were conducted with adults with RRMM who resided in the United States. In addition to the interviews, PRI was collected from YouTube and a patient advocacy website. Key themes from the interviews and PRI were summarized, and illustrative quotes were extracted.

**Results:**

Twenty participants were interviewed; 11 were female, and mean (standard deviation) age was 60 (7.0) years. The PRI included 14 posts and 19 unique contributors (10 were female). Similar treatment‐related symptoms were reported in the interviews and PRI. Fatigue and pain were the most frequently reported symptoms while receiving treatment in both the interviews and PRI. These symptoms had a meaningful impact on health‐related quality of life (HRQOL); being off treatment and returning to normal living was described as an ideal treatment outcome. Nearly all interview participants (*n* = 18) preferred a treatment that would allow for a treatment‐free interval, if it had the same efficacy and safety profile as a continuous treatment.

**Conclusion:**

The symptom experience reported in this study is consistent with known RRMM symptoms and HRQOL impacts. Additionally, this study highlighted that patients' treatment expectations are changing relative to their past treatment experience. Individuals living with RRMM strongly desire therapies with a treatment‐free interval and minimal impact on their HRQOL.

## 1 INTRODUCTION

Multiple myeloma (MM) is a malignancy of clonal plasma cells characterized by periods of relapse and remission. Relapsed refractory MM (RRMM) is a disease state that is nonresponsive or progressive on therapy.^1^, ^2^ In the absence of a curative treatment, attainment of remission is a sought‐after outcome,^3^ yet patients' remission periods often become shorter after each relapse.^1^ In addition, patients with RRMM commonly report symptoms such as pain, fatigue, lower physical functioning, and gastrointestinal issues, which can negatively affect health‐related quality of life (HRQOL).^4^, ^5^ Emerging therapies, such as chimeric antigen receptor T‐cell (CAR‐T) therapy, offer the possibility of not only maintaining but also improving patients' HRQOL.^6^, ^7^, ^8^ Patients with RRMM often require multiple lines of therapy, and with each line of therapy there are several important factors for patients to consider in a real‐world setting when choosing their treatment or therapy. Key factors of treatments include side effects and toxicity, convenience, financial costs, comorbid health conditions, and the influence of treatment on work and daily activities.^5^ Further, the complexity and heterogeneity around RRMM and in this patient population can lead to different treatment needs and expectations among patients.^5^ Many therapies are under investigation for MM, and considering these various treatment factors is critical for better understanding of patients' experiences, perceptions, and expectations of current and new treatments.

Patient‐reported information (PRI) shared on social media platforms can provide distinct and valuable insight into patients' disease and treatment experiences.^9^, ^10^ For example, patients may use common social media sites to share information about their health conditions, ask for advice, seek social support, and/or share knowledge.^11^, ^12^ Previous studies have shown that patients with diabetes and patients who have suffered a stroke have used social media as a resource for receiving social support, health information, and guidance from other patients with the same conditions.^13^, ^14^ Recent US (United States) Food and Drug Administration guidance has indicated that social media reviews are a useful addition to traditional research methods.^9^ Social media data are increasingly used in patient‐centered research to understand the experiences of rare patient groups who may share their experiences on patient advocacy websites and mainstream websites (e.g., YouTube).^15^, ^16^ This study explored individuals' experiences with RRMM and their perspectives on current and emerging treatments, including novel options such as CAR‐T therapy. This was a small pragmatic study that combined a qualitative interview and social media review approach to gain insights into patient experiences with treatment and therapies for MM using qualitative interviews and PRI shared on social media.

## 2 METHODS

### 2.1 Study design

Semistructured qualitative interviews were conducted among individuals with RRMM in the US. Interviews (approximately 60–90 min) were conducted via telephone or a web‐based meeting platform without video and facilitated by a semistructured interview guide, and field notes were collected. All interviews were audio recorded, transcribed, and conducted at the investigators' workplace. Additionally, a targeted social media review was conducted to collect PRI from individuals with RRMM. This study was conducted by both male and female researchers who had experience in qualitative research methods. Only the researchers and the participants were present when the interviews were being conducted.

### 2.2 Participant recruitment for qualitative interviews

Participants were recruited by a patient recruitment firm (Rare Patient Voice) using a study‐specific screener. The target study sample was 20 participants with RRMM, including a mix of individuals with and without prior CAR‐T treatment experience. Participants were eligible for the study if they were aged ≥18 years; had a self‐reported diagnosis of RRMM; were willing to participate in audio‐recorded qualitative interviews; were able to speak, read, and write English; and had access to a computer or tablet. No relationships with participants were established prior to study commencement.

### 2.3 Data collection

#### 2.3.1 Qualitative interviews

Interviews were conducted between November 7, 2019, and February 14, 2020, by researchers from RTI Health Solutions who have extensive experience in conducting qualitative interviews. At interview start, participants were reminded about the purpose and format of the interview as well as their rights as research participants, and verbal informed consent was obtained. The interviews were facilitated by a semistructured interview guide, which included a series of closed‐ended background sociodemographic and health questions, including MM disease characteristics and treatment experience. Participants were then asked a series of open‐ended questions about their symptom experience due to their RRMM and its treatment, the impact on their lives, and the extent to which these symptoms and impacts were important to them. A subgroup of participants without CAR‐T experience was asked additional open‐ended questions about their hypothetical perspectives on CAR‐T therapy. There were no repeat interviews carried out, and interview transcripts were not returned to the participants.

#### 2.3.2 Social media review

The social media review was conducted between April and May 2020. An initial pragmatic Google search using the terms “multiple myeloma,” “patient stories,” and “patient narratives” was performed to identify potential patient advocacy websites that hosted patient‐contributed content. Four patient advocacy sites of interest were identified (www.curetoday.com, www.cancercare.org, www.patientpower.info, and https://www.blogforacure.com). Following a review of patient narratives on each website, only Patient Power provided relevant PRI to address the study objective. A supplemental pragmatic review of YouTube using variations of the search terms “relapsed refractory multiple myeloma” and “CAR‐T experience” was conducted to identify relevant video content that included stories of patients with RRMM. Video data were collected from both YouTube and Patient Power. Blog data were collected from Patient Power only. Permission was granted from Patient Power to use their website content. YouTube is a global online platform where registered users share videos; videos with “public” privacy settings are publicly available. The target population included contributors with RRMM as determined by either a self‐reported diagnosis of RRMM or a self‐reported diagnosis of MM accompanied by reports of previous treatment(s) and disease progression while on treatment. Contributors who self‐reported having received CAR‐T therapy for MM were specifically targeted. During the social media review, data extraction was expanded to include commentaries on the prospect of CAR‐T therapy by contributors with RRMM who were CAR‐T therapy naïve. Key patient demographic and diagnostic characteristics were not always publicly available.

### 2.4 Data analysis

#### 2.4.1 Interview data

Participant characteristics were summarized. The interview transcripts were reviewed and deidentified prior to analysis, to maintain patient confidentiality and ensure transcript accuracy and completeness. The data underwent thematic analysis involving the extraction and review of excerpts related to the target areas addressed in the interview.^17^ Previous research has posited that a qualitative sample size of up to 20 participants is sufficient to establish concept saturation or the qualitative threshold at which no new topics emerge from the interview data.^18^, ^19^, ^20^, ^21^ Furthermore, the richness of the dialog has been considered an important factor for determining qualitative sample sizes; the quality of the interview data may be more salient than the number of subjects in qualitative research.^22^, ^23^

Key themes related to RRMM and treatment experiences were summarized, and illustrative interview quotes were used to support key findings identified from field notes. The thematic analysis was conducted using Atlas.ti 7 coding software (Atlas.ti). A mixed approach was applied, where the coding followed an iterative process whereby deductive coding was applied initially following a prescribed coding framework, with new codes added as themes and concepts emerged from the data. Newly emerged codes were retrospectively applied to earlier transcripts. Two different researchers coded each of the transcripts to ensure consistency in the coding.

#### 2.4.2 Social media data

Video footage and discussion blogs were manually reviewed to identify relevant content. Contributor narratives were thematically analyzed with descriptive codes developed based on themes identified in the data. Where available, demographic and clinical characteristics were extracted from the video and blog content.

## 3 RESULTS

### 3.1 Sample characteristics: qualitative interviews

Interviews were conducted with 20 participants; and when asked to give consent, none of the participants refused to take part in the interviews. The mean (standard deviation) participant age was 60 (7) years, and just over half (*n* = 11) were female. Most participants (*n* = 18) had at least some college education. Only two participants reported their general health to be “excellent” (*n* = 2), and one reported their general health to be “very good.” Most participants (*n* = 16) reported that they were affected “a lot” or “quite a lot” by their MM (Table 1). During the interviews, none of the participants requested to stop the interview prior to completion. Nineteen of the 20 interview participants had experience with intravenous (IV) infusions and oral medication, and most participants (*n* = 17) underwent a stem‐cell transplant. The most common treatments participants received for MM were bortezomib and lenalidomide (*n* = 19 for both), daratumumab (*n* = 16), and pomalidomide (*n* = 10). Participants also frequently reported steroid use, particularly dexamethasone (*n* = 18). Only one participant reported receiving radiation for MM. Less than half of participants (*n* = 7) previously participated in an MM clinical trial. One patient had experience with CAR‐T therapy, a 53‐year‐old White female, on long‐term sick leave or disability. The patient reported her general health as “poor” and that she had been affected “a lot” by MM.

**TABLE 1:** Interview participant demographics

Characteristic	Total (*n* = 20)
**Age (years)**	
Mean (SD), median	59.8 (6.5), 59
Range	48–72
**Gender, *n* (%)**	
Female	11 (55.0)
**Relationship status, *n* (%)**	
Married/living as married/civil partnership	12 (60.0)
Widowed	2 (10.0)
Divorced	4 (20.0)
Single	2 (10.0)
**Race, *n* (%)**	
White/Caucasian	17 (85.0)
Black/African American	1 (5.0)
Asian/Pacific Islander	1 (5.0)
Prefer not to answer	1 (5.0)
**Ethnicity, *n* (%)**	
Not Hispanic/Latino	19 (95.0)
Hispanic/Latino	1 (5.0)
**Employment status, *n* (%)**	
Working full time	1 (5.0)
Working part time	2 (10.0)
Retired	9 (45.0)
Long‐term sick leave or disability	8 (40.0)
**Education, *n* (%)**	
High school diploma	2 (10.0)
Some college or associate's degree	7 (35.0)
College graduate/bachelor's degree	6 (30.0)
Advanced or professional degree	5 (25.0)
**Health insurance, *n* (%)**	
Commercial insurance	5 (25.0)
Medicare, Medicaid, or public assistance program	8 (40.0)
Both private and public insurance	7 (35.0)
**General health, *n* (%)**	
Excellent	2 (10.0)
Very good	1 (5.0)
Good	6 (30.0)
Fair	7 (35.0)
Poor	4 (20.0)
**Affected by MM, *n* (%)**	
A lot	12 (60.0)
Quite a lot	4 (20.0)
A little	4 (20.0)
Not at all	0 (0)
**Other health conditions, *n* (%)**	
Yes	14 (70.0)
**CAR‐T therapy experience, *n* (%)**	
Yes	1 (5.0)

Abbreviations: CAR‐T, chimeric antigen receptor T‐cell; MM, multiple myeloma; SD, standard deviation.

### 3.2 Sample characteristics: social media review

Ten videos and four blogs were identified during the social media review, including 19 unique contributors with RRMM. Since PRI exists outside of the traditional research context, key demographic and disease characteristics were not always available. Of the 19 contributors, 4 reported their age (range, 51–72 years), 10 were female, and 9 were male. Half of the videos (*n* = 5) focused on one unique individual (female, aged 51 years); the four blogs also originated from this single contributor. Of the remaining five videos, four were contributed to by multiple individuals (three featured conversations; one included separate segments), and the remaining video detailed another individual contributor's experiences. Eight videos and the four blogs were published on the US‐based Patient Power website. The remaining two videos were uploaded to YouTube by Myeloma UK and Myeloma Canada, respectively. All social media sources were published on their respective platforms between August 2017 and November 2019, prior to the World Health Organization's declaration of the global coronavirus disease 2019 (COVID‐19) pandemic on March 11, 2020.

### 3.3 Symptom and disease impact experience

Concept saturation was achieved for RRMM symptom experience and HRQOL impacts, including physical functioning and activities, social functioning, relationships, work, sleep, and psychological/emotional impacts; over 90% of these key concepts were identified in the first 12 interviews. The interview sample was composed of patients with RRMM who, by the nature of their disease, had undergone numerous intensive therapies. Therefore, the patient‐reported symptom experience included a patchwork of MM and treatment‐related symptoms, as well as the consequences of participants' own compromised immune systems. Overall, interview participants reported experiencing similar key symptoms. Fatigue (*n* = 18) and pain (*n* = 16) were the most commonly reported. Fatigue (*n* = 5), cognitive dysfunction (*n* = 4), and pain (*n* = 3) were frequently reported as the most bothersome symptoms. Numerous additional, idiosyncratic symptoms were also reported by individual participants. However, the key symptoms that emerged from the interview data were confirmed by the PRI, notably pain, fatigue, fractures, fevers, and bone lesions. Symptom frequency, intensity, and impact on HRQOL all contributed to which symptom the interview participants considered the most severe and/or concerning. Although not reported by all participants, a worsening in perceived severity of symptoms, such as pain and fatigue, indicated the onset of relapse to some participants.

Both the interview data and the PRI highlight that MM had a debilitating impact on individuals' HRQOL (Table S1). For the interview participants, symptoms impeded their mobility (e.g., walking [*n* = 6], lifting things [*n* = 5]) and ability to carry out activities of daily living (e.g., shopping [*n* = 7], housework [*n* = 7], and gardening [*n* = 8]), as well as their leisure and work life (e.g., physical activity/sports [*n* = 11], work [*n* = 18]). Participants' social life and relationships were also disrupted by their MM symptoms. The emotional and psychological impact of relapsing was often more devastating for participants than their initial MM diagnosis. The PRI highlighted that the contributors' perceived loss of independence and control of their lives was a source of emotional distress. The inevitable need for individuals to adapt to a “new normal,” restructure life to accommodate doctor appointments and treatments, and the unpredictability of treatment success were key themes that emerged from the PRI. Although the new normal involved restrictions for contributors, it also redefined how they chose to live their lives; contributors made positive changes to their diets and lifestyles, sought out the benefits from a slower‐paced life, and prioritized spending quality time with family and friends. However, individuals' emotional and psychological distress was often exacerbated by the perceived failure of their treatments.

### 3.4 Perspectives on current MM treatment

Concept saturation was achieved for the key treatment‐related concept areas of treatment experience (i.e., impact on day‐to‐day life, treatment type preferences, treatment features, and treatment outcomes); almost 80% of these key treatment concepts were identified within the first eight interviews. Interview participants were generally happy with the treatments they received, despite the inherent disadvantages of continuous intensive treatments (i.e., inconvenience and/or side effects). Participants acknowledged that the treatments were effective at keeping them alive. The impact of treatments on participants' day‐to‐day lives varied by treatment type. Oral medications and subcutaneous injections had a minimal impact, other than potential side effects (e.g., fatigue and mood swings), while IV infusions were more burdensome (Table 2). PRI provided supportive evidence that treatment side effects (e.g., neuropathy and weakened immune systems) affected contributors' day‐to‐day lives. Many contributors experienced stress from continuous treatment and switching treatments to achieve remission but were grateful to experience long periods of clinical remission (Table S2).

**TABLE 2:** Interview participant treatment experiences and preferences (*N* = 20)

Key theme	Illustrative quotes
Treatment experience	*“The orals are more convenient to take because you just have them at home*…*the IV, I honestly don't mind it. I go in there* …*I actually don't mind going down there*… *they put it* [the IV line] *in, and I sit and read, so it's not too bad.”* (Female, 69 years)
Impact on day‐to‐day life	*“I go into the hospital for my IV treatment, which takes 6 to 7 hours*…*so that takes a day out. It takes the next day for me to rest up and recuperate. So, therefore, I cannot work a full‐time job. That's 2 days out of the week that I cannot work.”* (Male, 57 years) *“I actually enjoy doing some of my infusions because my nurses are wonderful…there's a group of Friday friends that make sure we have our appointments on Friday*…*they're funny, they're friends, they're upbeat. And I meet other people there that I've enjoyed a lot. So, it's kind of a social outing.”* (Female, 67 years)
Treatment type preferences	*“If there was a path to a straight oral, that would change the quality of my life dramatically because I wouldn't feel chained to an infusion.”* (Male, 58 years) *“Well here in America, if it's infused then you can get it covered by Medicare. If it's a pill, then you can't get it covered by Medicare or the private insurance pays for it, and then of course you have to pay 20%, which can be $1000, $1500 a month*…*so I would have to say infusions are better, but from a convenience perspective, of course it would be oral.”* (Female, 64 years)
Treatment features	*“I'm looking for the quality of life. The life that I have left, I want to live with quality. Not to be a burden on anybody. I want to be self‐sufficient, so quality.”* (Female, 59 years)
Treatment outcomes	*“Okay, to dream, because it's not going to happen, would be full remission*…*it will never be remission. But if you could create a state in me where I had progression‐free survival over a multiyear period, I would consider that to be a functional cure, even though it would inevitably come back.”* (Male, 58 years) *“Oh my God, that [to be off treatment] would be huge. If I could do that and maintain levels? Oh, that's a pipe dream, I had to give that up. That would be marvelous, that would be the holy grail, wouldn't it?”* (Male, 58 years)

Abbreviations: CAR‐T, chimeric antigen receptor T‐cell; IV, intravenous.

The possibility of being off treatment was a highly desirable outcome for many participants (*n* = 15), with one referring to it as “the holy grail” of treatment outcomes. Most participants (*n* = 18) preferred a treatment that would allow a treatment‐free interval compared with continuous treatment, if it had the same efficacy and safety profile. However, half of participants (*n* = 10) were skeptical that being off treatment was realistic. Participants were unsure whether such a treatment was possible and would no longer necessitate continuous treatment. Other important participant‐reported treatment features included treatment effectiveness, fewer side effects, improvement in HRQOL, and convenience. Two participants considered cost to be important, and seven participants considered delaying progression of RRMM, remission, and reducing or eliminating treatment side effects to be good treatment outcomes.

### 3.5 Perspectives on CAR‐T therapy

One interview participant and one social media contributor commented on their experiences with CAR‐T therapy. Both individuals exhausted all other therapies before progressing to CAR‐T therapy. The potential for sustained remission from CAR‐T was an attractive feature and a key factor in the interview participant's decision to undergo the treatment. The contributor described CAR‐T therapy as her “last hope.” The interview participant experienced initial success while participating in a CAR‐T clinical trial in 2018, but she was later discontinued from the trial due to severe side effects that resulted in a 29‐day hospital stay. While the participant reached partial remission due to the CAR‐T therapy, her remission progressively diminished, which she attributed to the severe side effect complications. The participant reported that she was extremely distressed with the overall outcomes; she was desperate to live life without continuous treatment and viewed CAR‐T as the only option to achieve this goal: “I think I'm just really crushed that [this]…happened to me. Because maybe I would still be off treatment and traveling and doing things I used to do.” Despite the participant's negative experience, she was eager to recommend CAR‐T therapy to other people: “the time you put in compared to what you can get out of it [CAR‐T therapy], it's miles…better than anything else.”

The social media contributor described her experience with CAR‐T as easy, quick, and better than stem‐cell transplantation and chemotherapy. Following CAR‐T therapy, she experienced her first complete remission and felt better immediately: “For me it was life changing, and lifesaving. A true scientific miracle come true…the impossible had finally happened. Where just a couple of months ago I was looking death in the eyes, I was now a cancer‐free patient.” She felt she was able to return to “normal life,” including work and being active. She stated that she felt healthier post CAR‐T than 2 years prior to the original diagnosis. Unfortunately, the contributor relapsed 1 year after receiving CAR‐T treatment; she was disappointed and devastated. The return of her MM was accompanied by the return of her symptoms (e.g., bone lesions and fractures). The contributor noted that she was faced with limited options for her next line of treatment.

Due to the difficulty recruiting individuals with CAR‐T experience, the final seven participants without CAR‐T experience were asked additional open‐ended questions about their perspectives on CAR‐T therapy. Most (*n* = 6) were familiar with the concept of CAR‐T therapy prior to the interview, and all participants who were familiar with the therapy had discussed it with their healthcare provider. While participants were hopeful that a cure for their RRMM was possible, they thought it was unrealistic based on current therapies. One participant commented on the possibility of a cure: “I mean, is it possible? Yes. I'm hoping in my lifetime.” Only one participant reported that she would not consider CAR‐T therapy; she felt it still needed further development and receiving CAR‐T therapy would make her ineligible for other clinical trials. Adverse events related to the therapy, including death, were key concerns for participants, and they reported that they would consider the effectiveness and previous success rate of CAR‐T therapy before undergoing the procedure. Two participants raised concern about the financial cost, and only one participant reported that he was aware of instances of relapse among CAR‐T patients (Table S3).

## 4 DISCUSSION

This study provided novel and valuable insight into patients' perspectives on current and emerging therapies for RRMM through qualitative interviews and a review of PRI. Fatigue and pain were the most common symptoms reported, and treatment‐related symptoms greatly affected individuals' day‐to‐day lives. Concept saturation for RRMM symptom experience, HRQOL impacts, and areas of treatment experience was achieved within the interview sample. This symptom and HRQOL experience is consistent with patient‐reported symptomatology and HRQOL impacts observed in previous research.^3^, ^24^, ^25^ Overall, distinctive themes emerged from both participant interviews and PRI that showed individuals with RRMM are still experiencing the same negative treatment impacts on their HRQOL but are also hoping for future therapies that will allow them to be off treatment. These findings indicate that patients' symptom experiences and HRQOL have remained consistent over the past 5 years,^3^, ^24^ despite the development of novel treatments.

Both the interview data and PRI showed that patients' treatment expectations were influenced by their previous treatment experiences and outcomes. Patients' treatment expectations fluctuated depending on the degree of perceived success or failure of previous treatments, adjusting from an initial idealistic perception of a curative treatment effect to pragmatic expectations (e.g., controlling symptoms or delaying disease progression). For many patients, the possibility of being off continuous treatment but with disease control or remission would be an ideal scenario; one participant referred to being off treatment as “the holy grail” of potential treatment outcomes, as it would allow them to return to normal living. Furthermore, patients held out hope for the development of a curative treatment for MM within their own lifetimes and are enthusiastic about therapies with a treatment‐free interval.

While participants understood the possibility of a single treatment with a treatment‐free interval and were receptive to the idea, they were skeptical that such a treatment existed. Their perceived benefits of a single treatment included the cessation of continuous treatment and the much sought‐after treatment‐free interval. For participants, this treatment‐free interval was analogous with lowered treatment burden and psychological impact, improved HRQOL, and, ultimately, a return to “normal life.” However, participants were concerned about the potential safety and effectiveness of such a therapy, the lack of published evidence on its previous success, and potential financial burden.

Significant improvements in certain patient outcomes (e.g., prolonged survival) have been made as a result of new therapeutic strategies,^26^, ^27^ but these findings suggest that less progress has been made to improve HRQOL. Prior research has identified HRQOL as an important treatment priority,^28^, ^29^ and this study found that individuals with RRMM desire therapies that not only maintain but also improve their HRQOL. New treatments (e.g., CAR‐T therapy) in development may help patients achieve these desired outcomes,^8^, ^30^, ^31^ but more research is needed once they are widely available. In this study, one of the two individuals who underwent CAR‐T therapy expressed her excitement at being able to return to “normal life” post treatment, and both experienced an improvement in their MM as a result of the treatment. Their experience is consistent with the findings from the published literature on clinical trials of CAR‐T therapy; clinically meaningful improvements in pain, fatigue, physical functioning, and global health status were seen in patients following treatment.^8^, ^30^, ^31^ CAR‐T therapy has also been shown to have less of an negative impact on patients' HRQOL compared with autologous or allogeneic stem‐cell transplantation.^30^, ^31^ The CAR‐T–specific findings in this study indicate that there is an unmet need to educate patients about the possibilities of these emerging CAR‐T therapies.

## 5 LIMITATIONS

This study has several limitations, including the self‐selecting nature of the recruitment process. Participants were recruited from an established recruitment panel or self‐reported their experience on social media, so it is likely that recruitment was biased toward patients who were interested in research and knowledgeable about RRMM. Social media data are also unregulated and may include irrelevant content that has questionable credibility and may also be subject to bias. Additionally, eligibility was determined based on self‐reported data, which may lead to misclassification associated with self‐reported disease identification. This study's small sample size, especially for patients with CAR‐T experience, and the higher levels of education within the sample may limit the generalizability of our findings. Although generalizability in qualitative research is not always an expected characteristic, it can be achieved through qualitative research methods by examining relationships among concepts and categories to gain knowledge insights.^32^

## 6 CONCLUSION

Despite these limitations, this study provides much‐needed insight into individuals' preferences for and experiences of current and emerging RRMM treatments. Our findings suggest that the symptom experience of individuals living with RRMM has remained consistent over the past 5 years and that treatment‐related symptoms can have detrimental impacts on patients' HRQOL. However, this study also found that individuals living with RRMM are hopeful for a cure in their lifetime and view being off treatment as the ultimate priority for new therapies.

## CONFLICT OF INTEREST

Rebecca Crawford, Ross Morrison, and Lynda Doward are employees of RTI Health Solutions, and this study was performed under a research contract between RTI Health Solutions and Janssen Pharmaceuticals Inc. Katharine S. Gries, Satish Valluri, John Fastenau, Tzu‐Min Yeh, Yunsi Olyslager, Jenna D. Goldberg, Jordan M. Schecter, Carolyn C. Jackson, and William Deraedt are employees of Janssen Pharmaceuticals Inc.

## AUTHOR CONTRIBUTIONS

**Rebecca Crawford:** Conceptualization (equal); data curation (equal); investigation (equal); project administration (equal); supervision (equal); writing – original draft (equal); writing – review and editing (equal). **Katharine S Gries:** Conceptualization (equal); supervision (equal); writing – review and editing (equal). **Satish Valluri:** Conceptualization (equal); supervision (equal); writing – review and editing (equal). **John Fastenau:** Conceptualization (equal); supervision (equal); writing – review and editing (equal). **Ross Morrison:** Conceptualization (equal); data curation (equal); investigation (equal); project administration (equal); writing – review and editing (equal). **Tzu‐Min Yeh:** Writing – review and editing (equal). **Yunsi Olyslager:** Writing – review and editing (equal). **Jenna D Goldberg:** Writing – review and editing (equal). **Jordan M Schecter:** Writing – review and editing (equal). **Caorlyn C Jackson:** Writing – review and editing (equal). **William Deraedt:** Writing – review and editing (equal). **Lynda Doward:** Conceptualization (equal); supervision (equal); writing – review and editing (equal).

## ETHICS STATEMENT

The RTI International institutional review board approved the qualitative interviews (STUDY00020872) and determined that the social media review did not constitute research with human subjects (STUDY00020936).

## Supporting information


**Table S1** Interview Participants' Health‐Related Quality of Life Impact of Multiple Myeloma (*N* = 20)
**Table S2.** Social Media Contributor Treatment Experiences and Preferences (*N* = 19)
**Table S3.** Perspectives on CAR‐T Therapy From Participants Without CAR‐T Therapy Experience (*N* = 7)

## Data Availability

The data sets generated and/or analyzed during the current study are available from the corresponding author upon reasonable request.
