# Supplementary material for: The patient experience of relapsed refractory multiple myeloma and perspectives on emerging therapies
Source: Cancer Rep (Hoboken). 2022 Feb 15;5(11):e1603. doi: 10.1002/cnr2.1603 (PMC9675381; doi:10.1002/cnr2.1603)
Supplement: Supplementary file 1 — Table S1 Interview Participants' Health‐Related Quality of Life Impact of Multiple Myeloma (N = 20) Table S2. Social Media Contributor Treatment Experiences and Preferences (N = 19) Table S3. Perspectives on CAR‐T Therapy From Participants Without CAR‐T Therapy Experience (N = 7) [file CNR2-5-e1603-s001.docx]

Supporting Information, Table S-1. Interview Participants’ Health-Related Quality of Life Impact of Multiple Myeloma (N=20)

| Key Theme | Illustrative Quotes |
| --- | --- |
| Physical functioning and activities (e.g., walking [n = 6], lifting things [n = 5], difficulty with self-care [n = 1], shopping [n = 7], housework [n= 7], gardening [n = 8], physical activity/sports [n = 11], travel [n = 14]) | *“It’s too much for me to be able to walk from, to the gate. And so I have to realize that. And whatever activities we’re going to be doing on vacation, I have to make sure that I can have a wheelchair.”* (Female, 63 years) |
| Sleep (n = 6) | *“My family doctor gives me a sedative so I can go to sleep. Otherwise I’d never fall asleep*.” (Male, 56 years) |
| Work (n = 18) | *“I was a baker, actually, and I had to give it up…It’s like having my heart ripped out. It was just another thing that I really loved that was taken from me. It’s one of the things that I haven’t reconciled with because that was what I loved.”* (Male, 58 years) |
| Social functioning (n = 10) | *“When you get invited to various social gathering as a family and there are times when I am just too exhausted to go. Or it’s occurring right after my chemo and there’s just, like, no way. I just need to sleep. And then there’s this third piece which is if I…there’s just times I’m not up to chatting casually with people. Because I just don’t know what I would say. I just don’t want to have casual conversations with people.”* (Female, 53 years) |
| Relationships (n = 7) | *“I don’t see my grandkids as much as I used to and they don’t even live a mile away, and they’re that close. But I used to spend an afternoon with them. After an hour, I’m ready to leave. I don’t babysit at my house like I used to. I just get too tired. I don’t have the energy to do the things that I want to do.”* (Female, 65 years) |
| Psychological/emotional (e.g., frustration [n = 8], sadness [n = 8], anger [n = 5], anxiety [n = 3], concern about infections [n = 15], concern about fractures [n = 6], feeling depressed [n = 10], fear of mortality [n = 6], loss of independence [n = 2], feeling trapped [n = 3]) | *“It’s always keeping in mind that you have this illness. You really can’t move on with your life the way that you would normally have. You just sort like…It sort of has you in a prison*.” (Male, 68 years) |

Supporting Information, Table S-2. Social Media Contributor Treatment Experiences and Preferences (N=19)

| Key Theme | Illustrative Quotes |
| --- | --- |
| Treatment experiences | *"…combination therapy was able to put my myeloma in what’s called a very good partial remission. But it took almost 3 years to get that maximum response."* (ID11_Female)  *"I had come to realize that although chemo had kept me alive for 5 years, it was also slowly destroying my body. I went through those 13 different forms of treatment at a phenomenal pace and I felt my body slowly decline. Even though all these different forms of treatments had kept me alive for 5 years, both the cancer and the treatments were slowly killing me."* (ID01_Female)  *"[Post–stem-cell treatment] I knew in the back of my mind that it would return as it always does return, but nobody could say exactly when. When it came back, I wasn't totally surprised, I was a bit disappointed because I knew I would have to start there the grind again cycles of medication all this kind of stuff."* (ID02_Male)  *"…we knew it wasn't a cure and I was very fortunate and felt very pleased to have got 5 and a half years out of it."* (ID05_Female) |
| Treatment decision drivers | *"…after relapsing from my 13th line of treatment, I told [name deleted] I was done with chemo. It wasn't that I wanted to die, something that would happen for sure without treatment and likely very fast. No, I had come to realize that although chemo had kept me alive for 5 years, it was also slowly destroying my body."* (ID01_Female)  *"…one of things that I was considering back then was how the treatment was given. And one of the treatments that I chose was an oral treatment because that allowed me to continue to be employed."* (ID11_Female)  *"…side effects probably would be the least important. I think I can probably endure a lot at this point in my life if it's just temporary. I do have three businesses, and that would come into play as far as how treatments would go… I would be more willing to trust something that had a long-term track record of success than something new that we really just don’t know that much about."* (ID08_Male) |

Supporting Information, Table S-3. Perspectives on CAR-T Therapy From Participants Without CAR-T Therapy Experience (N=7)

| Key Theme | Illustrative Quotes |
| --- | --- |
| Familiarity with CAR‑T treatment | *“Oh, I know it’s very similar to the stem cell. You go in and they spin your cells out, and then they send them off to a lab. And 2 and a half weeks later or so, when they get all the material back, they put it back into your system.”* (Male, 72 years) |
|  | *“The couple of conversations that we’ve had about it, my doctor feels that the CAR-T is in the Hyundai phase, and he would prefer to be in the Cadillac phase before I get it.”* (Female, 49 years) |
| CAR‑T therapy considerations | *“The other thing is that we feel that there are other treatments right now that I can still continue to try while the CAR‑T is getting cleaned up a little bit and having better success. And the third thing that we’ve discussed is that if I have the CAR‑T, it eliminates me from being eligible for clinical trials, and so we’re kind of trying to stay away from CAR‑T so that I’m still eligible in the meantime for trials that might come about.”* (Female, 49 years) |
| CAR-T concerns | *“Yeah, it could kill me. It’s dangerous; no one knows what will happen.”* (Male, 58 years)  *“To be honest, I’ve heard it’s quite a doozy. I’ve heard it’s a hard…it’s much, much harder to go through than a regular stem-cell transplant. I’ve heard that there’s neurological problems. I’ve heard there’s a lot of ICU stays. I’ve heard that it’s pretty tough.”* (Female, 49 years) |
| Decision drivers | *“I think for me, it needs to have a better track record. They need to see more success coming out of it.”* (Female, 49 years) |
|  | *“I know the cost is the issue that people are talking about already. So the cost factor if it wasn’t on a trial, that may be prohibitive because it’s very high right now.”* (Male, 72 years) |
|  | *“The duration of its usefulness. Just the patient that I’m familiar with who have been [on] CAR-T, it seems people are relapsing within a year or so.”*  (Male, 58 years) |

CAR‑T = chimeric antigen receptor T‑cell.
